# Investigation of a Novel Piezoelectric Harvester for Capturing Rotational Motion

**DOI:** 10.3390/mi17020255

**Published:** 2026-02-16

**Authors:** Junxiang Jiang, Heming Wang, Liang Wang

**Affiliations:** 1 School of Mechanical and Civil Engineering, Jilin Agricultural Science and Technology College, Jilin 132101, China; wangheming@jlnku.edu.cn; 2 School of Mechanical Engineering, Northeast Electric Power University, Jilin 132012, China; wliang2021@neepu.edu.cn

**Keywords:** energy harvester, piezoelectric, frequency band, power

## Abstract

Piezoelectric energy harvesting technology has received great research interest in recent years. To harvest energy from rotational motion, this work proposes a cantilevered piezoelectric energy harvester based on an adjustable rigid parallel connection. The baffle was designed as a carrier for the rigid connection of the piezoelectric beams A, B and C. The theoretical model of the device was established, and equations for voltage and power were derived. The calculated intrinsic frequencies of the piezoelectric beams are consistent with the experimental results. The baffle size, the distance from the baffle to the free end, and the number of rotor bumps were used as variables in the experiments. The experimental results show that the proposed piezoelectric energy harvester can harvest energy across multiple frequency bands. The maximum average power of the proposed piezoelectric energy harvester is 110.49 mW at a load resistance of 10 kΩ and a rotational speed of 240 r/min. The maximum average power of the harvester is 36.44 mW at a load resistance of 10 kΩ and a rotational speed of 60 r/min. The rigid parallel connection not only broadens the energy harvesting bandwidth but also enhances the output performance of the harvester.

## 1. Introduction

In recent decades, the environmental climate has changed due to overexploitation of energy. Climate change and various natural disasters have become common problems for human beings. A growing number of scientists and scholars have started to devote themselves to environmental monitoring research [1,2,3]. Monitoring devices require an energy supply. The majority of these devices currently use batteries and solar energy for energy conversion to power their sensors due to the operating environment. Dry batteries need to be replaced regularly and are more polluting to the environment, so they are less commonly used [4,5]. In addition, solar energy can also be affected by factors such as light, temperature, and weather. Therefore, piezoelectric energy, frictional energy and electromagnetic energy have received a lot of attention in the collection of new energy sources [6,7]. Piezoelectric energy harvesting technology is considered for the energy supply of microdevices due to its simple structure, high conversion efficiency, and little impact on electromagnetic fields [8,9,10].

Not only high-frequency energy but also low-frequency energy is present in the environment. It will be difficult to adapt to the needs of outdoor applications if the piezoelectric energy harvester has a narrow collection bandwidth [11,12,13]. Therefore, the frequency bandwidth of the energy harvester needs to be extended to improve its output performance [14,15,16]. Piezoelectric energy harvesters are expected to be more widely applied for outdoor energy harvesting.

In most previous studies, cantilevered energy harvesters have been studied extensively. A large number of researchers have made significant contributions to the field of wide bandwidth and high power in energy harvesters. For example, Yang et al. [17] proposed a cantilever piezoelectric energy harvester for a wearable device with random vibration input; this smart wristband-sized energy harvester collects approximately 50 μW of root-mean-square power from arm movements. Usharani et al. [18] proposed a new piezoelectric patch cantilever beam with a stepped cross section for high-performance energy harvesting. Raju et al. [19] proposed a novel geometric cantilevered beam structure consisting of a tapered section with a rectangular cavity to achieve high power in the low operating frequency range. The proposed harvester with a cavity in the tapered portion provided 157.8% higher voltages compared to a continuous beam. Li et al. [20] proposed a wide-band piezoelectric harvester based on a cantilever beam, where the maximum output power of this energy harvester is 0.75 mW when the resistance is 510 kΩ and the frequency is 11 Hz. Chen et al. [21] presented a paper on a cantilevered piezoelectric energy harvester with a tunable function. Xie et al. [22] proposed an asymmetric monostable dual-cantilever structure consisting of a generating piezoelectric cantilever beam and an auxiliary cantilever beam; the proposed harvester with magnetically coupled asymmetric monostable provided higher voltage output with lower strain levels compared to a symmetric bistable beam. Tang et al. [23] proposed a cantilevered piezoelectric bimorph beam with a distributed end mass offset at the free end, in which a novel dynamic magnifier at the elastically restrained end was designed to widen the frequency bandwidth and enhance the power output from ambient vibrations.

Rotating machinery, ocean waves, wind power, etc., can provide rotational motion energy. Many scholars have conducted extensive research on the application of the piezoelectric effect to harvest rotational motion energy. These studies have improved the output efficiency of the energy harvester to some extent. Liu et al. [24] proposed a low-frequency non-contact rotary piezoelectric energy harvester excited by magnetic coupling, in which the piezoelectric plates are arranged in a cylindrical array along the axis; the device generates a maximum output power of 140.45 mW. He et al. [25] proposed a multi-group, dual piezoelectric energy harvester driven by inertial wheel with magnet coupling and plucking, in which the RMS voltage and power of the rectangular piezoelectric patch are 10.03 V and 0.80 mW at 550 r/min when the resistance is 100 kΩ. Kan et al. [26] proposed a wheel-type cantilevered piezoelectric rotational energy harvester for harvesting rotational motion energy, in which the rotary speeds, proof mass and piezo-cantilever mass, number of exciting magnets and so on affect the output characteristics of the system. Liu et al. [27] proposed a low-speed rotary piezoelectric energy harvester using a motion-based non-contact pendulum structure; the output power of this device is 0.0187 W when the resistance is 20 kΩ. Heidari et al. [28] proposed a new piezoelectric energy harvesting method using a buckled beam, in which two piezoelectric harvesters are placed inside the buoy in horizontal and vertical directions to collect the rotational motion of water particles on the sea surface. Su [29] proposed a rotationally excited piezoelectric–electromagnetic energy harvester with a lever structure, in which the piezoelectric energy harvester part generated a maximum of 1.04 mW when the resistance is 10 kΩ.

The indirect contact mode of magnetic excitation tends to affect output performance due to the magnetic field, temperature and other factors. Different from other structures, this study proposes a cantilevered piezoelectric energy harvester based on an adjustable rigid parallel connection for harvesting rotational motion energy. This piezoelectric energy harvester focuses on whether rigid parallel connections can improve output performance. This work investigates the effects of key structural parameters (baffle size, baffle position, and number of rotor bumps) on the output performance of the energy harvester through experimental comparison, aiming to verify the feasibility and superiority of the rigid parallel connection structure, rather than performing formal mathematical optimization. The experimental results show that the piezoelectric energy harvester can collect low and high-frequency energy effectively. Through the comparison of several power experiments, the peak power of the energy harvester can reach 110.49 mW. The innovation of this work is the use of rigid connections to efficiently distribute the impact force of the rotor to different piezoelectric beams. In conclusion, the proposed piezoelectric energy harvester has high output power and can also be considered for harvesting natural energy sources, such as wind and water energy. In the experimental study, a stepper motor was used to provide rotational energy. Natural rotational movements usually have unstable speeds and variable frequencies. However, the adjustable rigid parallel structure can adapt to a wide frequency band and can efficiently output at both low and high speeds. In practical applications, rotational motion energy can come from rotating components of household appliances, rotor hubs of ceiling fans, pump shafts, rotating hubs of bicycle wheels, etc. The study in this work will provide insights and information for subsequent research. The structure of this work is as follows: Section 2 presents the structural design and the working principle. Section 3 shows the development and analysis of the theoretical model. Section 4 details the experiments conducted and the discussion of the experimental results. Section 5 presents the conclusions drawn throughout the work.

## 2. Structure and Principle

**Figure 1:**
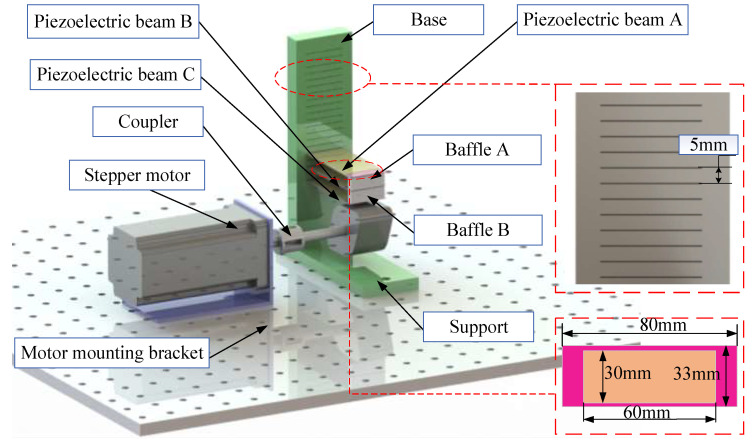
Schematic diagram of the proposed piezoelectric energy harvester.

The piezoelectric energy harvester proposed in this work mainly consists of a base, three rectangular piezoelectric patches (piezoelectric beams), two baffles, and a rotor. The parts other than the piezoelectric patches are fabricated by 3D printing. The overall structure of the cantilevered piezoelectric energy harvester is shown in Figure 1. The base is fixed on the platform, and one end of the base consists of multiple notches with a spacing of 5 mm. The size of the piezoelectric beam is 80 mm × 33 mm × 0.2 mm. The size of the ceramic piece in the middle is 60 mm × 31 mm × 0.2 mm. One end of the piezoelectric beam is embedded into the notches of the base as the fixed end, and its other end is made to contact the free end of the baffle. The notches at different positions are designed to adjust the spacing between the piezoelectric beams. Two gaps are reserved in the middle of the three piezoelectric beams to accommodate the baffles. Also, the baffles are available in various dimensions.

**Figure 2:**
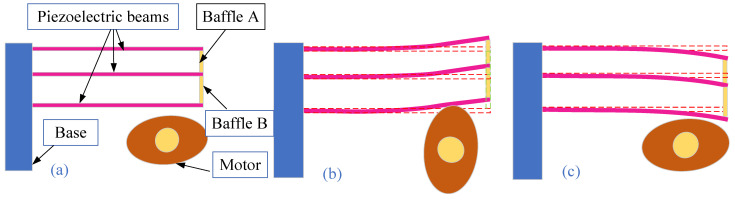
Working principle of the piezoelectric energy harvester. (**a**) Initial static phase, (**b**) excitation deformation phase, and (**c**) vibration rebound phase.

The working principle of the proposed piezoelectric energy harvester is shown in Figure 2. The working process of the piezoelectric energy harvester is divided into three main stages. In the initial stage, the rotor of the piezoelectric energy harvester is not in contact with the piezoelectric beam. The piezoelectric beam is in a slightly deformed state under its own gravity and the gravity of the baffle. The initial state of the energy collector is shown in Figure 2a. At this time, the output voltage of the energy harvester is weak. As the rotor continues to rotate, it applies continuous dynamic loads to the piezoelectric beams, causing the piezoelectric material to undergo periodic stress changes, thereby generating electrical energy. The protruding block on the rotor starts to squeeze the free end of piezoelectric beam C as the rotor rotates. The free end of piezoelectric beam C is displaced upward under the pressure from the rotor. The piezoelectric beams A, B and C are connected by means of baffles, through which the impact force of the rotor is transmitted. The deformation of the piezoelectric beam reaches its maximum when the raised block of the rotor is aligned horizontally with the piezoelectric beam. The output power of the energy harvester is maximum under this condition. The dimensions of the baffles can be changed, so the deformation of piezoelectric beam A differs from that of piezoelectric beams B and C when the dimensions of baffles A and B are not the same. The excitation deformation phase of the piezoelectric energy harvester is shown in Figure 2b. As the rotor continues to rotate, the protruding block moves away from the piezoelectric beam. At this time, the piezoelectric beam starts to bend downward after losing its support force under the combined action of its own gravity and elastic potential energy. The piezoelectric beam generates continuous vibration. The vibration rebound phase of the piezoelectric energy harvester is shown in Figure 2c. This completes the first working cycle of the piezoelectric energy harvester.

## 3. Theory and Analysis

**Figure 3:**
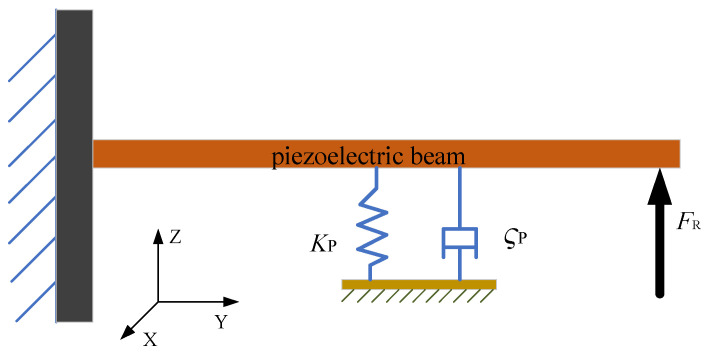
Analysis schematic of a single piezoelectric beam.

The rigidly connected cantilevered piezoelectric energy harvester can be simplified to a single-degree-of-freedom model. The simplified model is shown in Figure 3. The dynamic analysis of the system is carried out. The kinetic energy of the system includes the kinetic energy of the rotating motor *T_r_* and the kinetic energy of the rigidly parallel cantilevered beams *T_l_*.

(1) $$T=T_{r}+T_{l}$$

The kinetic energy of the rotating motor *T_r_* is

(2) $$T_{r}=\frac{1}{3}m_{r}r^{2}{\dot{\alpha }}^{2}$$

where *m_r_* is the mass of the rotating motor, *r* is the radius of rotation, and *α* is the angular displacement, which is correlated with motor speed.

The kinetic energy of a rigid parallel cantilevered piezoelectric energy harvester includes the kinetic energy of the substrate, the kinetic energy of the piezoelectric plate and the kinetic energy of the baffles.

(3) $$T_{l}=\frac{1}{2}\int_{V_{c}}\rho_{c}u^{t}\dot{u}dV_{c}+\frac{1}{2}\int_{V_{e}}\rho_{e}u^{t}\dot{u}dV_{e}+\frac{1}{2}\int_{V_{d}}\rho_{d}u^{t}\dot{u}dV_{d}$$

where *u*(*z*, *t*) is the transverse displacement vector; *V_c_*, *V_e_* and *V_d_* represent the volumes of the substrate, piezoelectric plate and baffle; and *ρ_c_*, *ρ_e_* and *ρ_d_* represent the densities of the substrate, piezoelectric plate and baffle. The kinetic energy of the whole system is

(4) $$T=\frac{1}{2}\int_{V_{c}}\rho_{c}u^{t}\dot{u}dV_{c}+\frac{1}{2}\int_{V_{e}}\rho_{e}u^{t}\dot{u}dV_{e}+\frac{1}{2}\int_{V_{d}}\rho_{d}u^{t}\dot{u}dV_{d}+\frac{1}{3}m_{r}r^{2}{\dot{\alpha }}^{2}$$

If only the polarization along the vertical direction is considered, the potential energy of the piezoelectric beam *E_b_* can be expressed as [21]

(5) $$E_{b}=\int_{V_{p}}\left(\frac{1}{2}E_{z}d_{3}+d_{3}\frac{\partial \lambda }{\partial z}\right)dV_{p}$$

where *λ* is the potential of the upper and lower parts of the electrodes of the piezoelectric ceramic layer, *E_z_* is the electric field along the vertical (*z*) direction, *V_p_* represents the volume of piezoelectric beam, and *d*_3_ is the electrical displacement of the energy harvester structure.

The energy harvester is simplified to a rigid body composed of three interconnected piezoelectric beams. According to Newton’s second law, the dynamic equations of the system can be obtained as follows [30]:

(6) $$K_{p}(t)+\delta V(t)+F_{R}=M_{p}\ddot{W}(t)+\varsigma_{p}\dot{W}(t)+K_{p}W(t)$$

where *M_p_* denotes the equivalent mass of the piezoelectric beam component with the baffle, *K_p_* represents the equivalent stiffness of the piezoelectric beam part, and *ς_p_* represents the equivalent damping of the piezoelectric beam part. *δ* is the electromechanical coupling coefficient of the piezoelectric ceramic (PZT). *K* is the correction factor of the amplitude. *p*(*t*) denotes the frequency of vibration received by ambient vibrations. In this structure, the base is fixed. *F_R_* is the vertical component of the exciting force generated by the rotating motor, *W*(*t*) represents the displacement of *M_p_*, and *V*(*t*) is the output voltage of the piezoelectric ceramic. The vertical excitation force generated by the rotor is the core force acting on the cantilever beam. The efficiency and distribution of force transmission between the piezoelectric beams A, B, and C depend on the structural parameters of the energy harvester.

The four variables *M_p_*, *K_p_*, *ς_p_* and *δ* are calculated as follows:

(7) $$M_{p}=M(i)+33m/140$$

where *m* represents the mass of the piezoelectric beam and *M*(*i*) represents the mass of the tip section (i.e., the mass of the two baffles). The calculation equation is

(8) $$M(i)=\rho_{d}\;l_{d}\;w_{d}\;t_{d}$$

where *l_d_*, *ρ_d_*, *t_d_* and *w_d_*, represent the length, density, height and width of either baffle.

(9) $$K_{p}=\frac{6E_{e}\;J}{{l_{c}}^{2}(2l_{c}+1.5l_{d})}$$

where *E_e_* is the elastic modulus of the piezoelectric ceramic, *l_c_* is the length of the piezoelectric beam substrate, and *J* is the rotational inertia, which is calculated as

(10) $$J=2\left[\frac{w_{c}{t_{e}}^{3}}{12}+w_{c}t_{e}{\left(\frac{t_{e}+t_{c}}{2}\right)}^{2}\right]+\frac{E_{c}w_{c}{t_{c}}^{3}}{12E_{e}}$$

where *E_c_* is the elastic modulus of the cantilever beam substrate, *t_c_* and *w_c_* are the thickness and width of the piezoelectric beam substrate, and *t_e_* is the thickness of the piezoelectric ceramic.

(11) $$\varsigma_{p}=2M_{p}\;\varepsilon_{b}\;f_{b}$$

where *ε_b_* is the mechanical damping ratio and *f_b_* is the intrinsic frequency of the piezoelectric beam.

(12) $$\delta =e_{31}\gamma_{b}\;w_{c}\frac{t_{c}+t_{e}}{2}$$

where *e*_31_ is the piezoelectric coefficient, *γ_b_* is the spatial derivative of the mechanical mode, and *w_c_* is the width of the piezoelectric beam substrate. The key material parameters used in the experiments are shown in Table 1.

According to Kirchhoff’s first law, the circuit equation of the energy harvesting circuit is obtained as follows:

(13) $$C_{P}\dot{V}(t)+\frac{\dot{V}(t)}{R_{1}}+\delta \dot{W}(t)=0$$

where *R*_1_ is the equivalent resistance of the piezoelectric ceramic and *C_P_* is the piezoelectric ceramic capacitance.

The intrinsic frequency of the piezoelectric beam *f_b_* can be obtained by the following equation since the model can be considered as a single-degree-of-freedom system [31]:

(14) $$f_{b}=\frac{1}{2\pi }\sqrt{\frac{3K_{J}}{l^{3}M_{P}}}$$

where *l* represents the length of the beam and *K_J_* represents the flexural rigidity of the composite cantilever.

The open-circuit voltage of a rigidly connected cantilevered piezoelectric energy harvester *V* under the rotor impact force is [32]

(15) $$V=\frac{3\frac{t_{c}}{t}(1-\frac{t_{c}}{t})\frac{Y_{s}}{Y_{m}}e_{31}l_{e}}{{\beta tw}_{e}}\left[F_{R}+M_{P}g\right]$$

(16) $$\beta =-2\frac{t_{c}}{t}\left(2{\left(\frac{t_{c}}{t}\right)}^{2}-3\frac{t_{c}}{t}+2\right)\left(1-\frac{Y_{s}}{Y_{m}}\right)+{\left(\frac{t_{c}}{t}\right)}^{4}{\left(1-\frac{Y_{s}}{Y_{m}}\right)}^{2}+1$$

where *t* is the thickness of the piezoelectric beam, *l_e_* is the length of the piezoelectric ceramic, *Y_s_* denotes the Young’s modulus of the copper substrate, and *Y_m_* is the Young’s modulus of the piezoelectric material. It can be concluded that the output voltage of the system is related to the dimensions of the piezoelectric ceramic and to the impact force of the rotor.

The impact force *F_R_* applied to the free end of the piezoelectric beam is related to the output voltage. Thus, the voltage generated by the energy harvester is a key factor in measuring its performance. The output voltage is positively correlated with the rotor impact force, and the variations in the output voltage observed in the experiment can be used to infer the variations in the force applied to the cantilever beam.

Then the expression of the output power of the piezoelectric energy harvester *P* is

(17) $$P=\frac{V^{2}R_{0}}{{\left(R_{0}+R_{1}\right)}^{2}}$$

where *R*_0_ is the load resistance. The supplementary parameters for Equations (13), (15) and (17) are shown in Table 2.

The kinetic energy of the piezoelectric beam is modeled using Newton’s second law, which in turn leads to the derivation of its potential energy. Since the model is considered a single-degree-of-freedom system, the frequencies are obtained. Finally, the output voltage and output power calculation equations of the energy harvester are established.

## 4. Experiment and Discussion

### 4.1. Experiment

**Figure 4:**
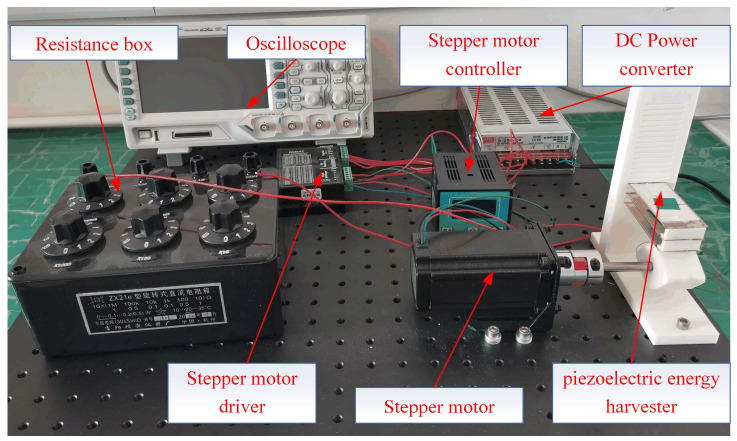
Test system for the piezoelectric energy harvester.

The experimental system is shown in Figure 4. The whole test system mainly includes a resistance box (ZX21e Fu yang, Hangzhou, China), an oscilloscope (DS1104Zplus RIGOL Suzhou, China), a DC power converter (S-250-24V, Xiezhou Electric Co., Ltd., Wenzhou, China ), a stepper motor (57BYG113-360A, Xiezhou Electric Co., Ltd., Wenzhou, China), a stepper motor controller (DKC-Y110, Xiezhou Electric Co., Ltd., Wenzhou, China), a stepper motor driver (DM542, Xiezhou Electric Co., Ltd., Wenzhou, China) and a self-made piezoelectric energy harvester. A prototype was made using 3D printing technology for experimental tests.

**Figure 5:**
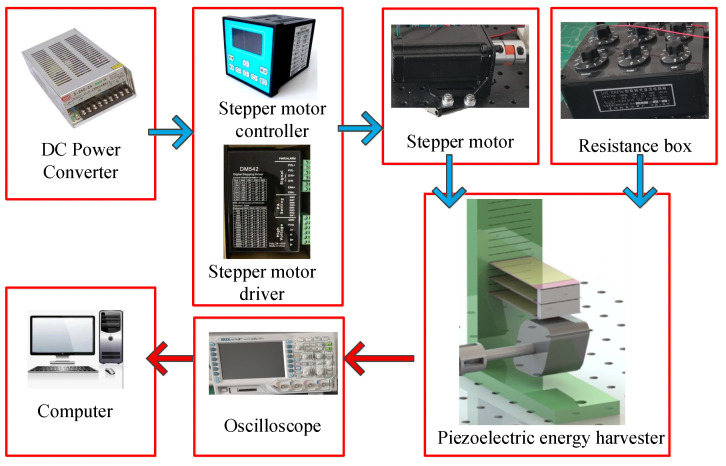
The connection relationship of the experimental device.

The connection relationship of the experimental device is shown in Figure 5. During the construction of the experimental system, the design and printing of the piezoelectric energy harvester are completed first. The harvester needs to be debugged multiple times during the assembly process to ensure that the piezoelectric beams and the base form a tight fit. The baffles between different piezoelectric beams are also made using 3D printing technology and are used to transfer the external excitation force to different piezoelectric beams. The piezoelectric energy harvester and stepper motor are bolted to the honeycomb plate.

The different devices are connected to their respective interfaces via wires. Thus, a system for driving and monitoring the electrical signals of the piezoelectric energy harvester has been established. This system can be used to monitor all types of rotating energy harvesters and is not limited to the rigid connection-based piezoelectric energy harvesters mentioned in this manuscript. The comparison of piezoelectric energy harvesters’ parameters is for performance verification rather than for mathematical optimization.

**Figure 6:**
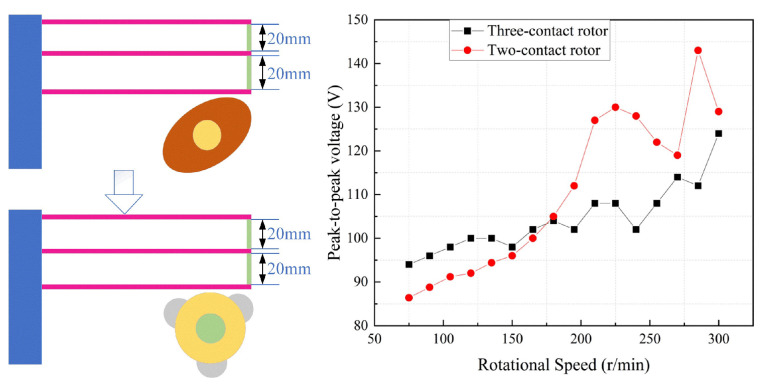
Voltage versus time for two-contact rotor and three-contact rotor for baffle sizes of 20 mm and 20 mm.

A stepper motor is used as an external power source for the energy harvester to simulate ambient mechanical rotational energy. To investigate the effect of the rotor over-angle on the performance of the energy harvester, a two-contact rotor and a three-contact rotor were selected. The comparative experiments with different rotors are shown in Figure 6. The over-angle of the three-contact rotor is larger than that of the two-contact rotor. The selected baffle sizes are 20 mm and 20 mm. The speed range is 60–300 r/min. The experimental results show that the output voltage of the selected piezoelectric energy harvester increases with speed. The voltage of the energy harvester with a three-contact rotor was higher when the speed was below 180 r/min. The voltage of the energy harvester with a two-contact rotor was higher when the speed was between 180 and 300 r/min. The excess angle of the two-contact rotor is smoother, while the three-contact rotor has a greater curvature of the excess angle. At low rotational speeds, the curvature of the contacts has less effect on the output of the energy harvester. This shows that the three-contact rotor exhibited an advantage at low speeds. The excessively rounded rotor is more suitable for the subsequent practical application of rigidly connected energy harvesters due to the variations in the external environment. Therefore, the rotor selected for the piezoelectric energy harvester was determined to be a two-contact rotor.

For the two-contact rotor, it induces two excitations with the piezoelectric cantilever beams per full rotation. The force per contact is more concentrated. For the three-contact rotor, it induces three excitations per full rotation, so the excitation frequency per unit time is higher. As shown in Figure 6, the peak voltage of the two-contact rotor (red curve) is generally higher than that of the three-contact rotor (black curve). This is because the concentrated force of the two-contact mode induces larger deformation of the piezoelectric structure. The voltage of the three-contact rotor fluctuates more gently due to more continuous excitations, while the two-contact rotor shows more obvious voltage fluctuations. We can derive preliminary general trends based on the 2-contact and 3-contact rotor results. N represents the number of contact rotors. Within a reasonable range (N is 2 or 3), increasing N increases the excitation frequency per rotation, which is beneficial for low-speed scenarios by compensating for low impact frequency. However, the impact force per contact decreases with N, leading to reduced piezoelectric beam deformation and lower peak voltage at high speeds. For N ≥ 4 (inferred), the excitation frequency continues to increase, but the trade-offs (uneven force distribution, increased friction, and manufacturing difficulty) become more prominent, leading to a decline in overall power output.

### 4.2. Results and Discussion

**Figure 7:**
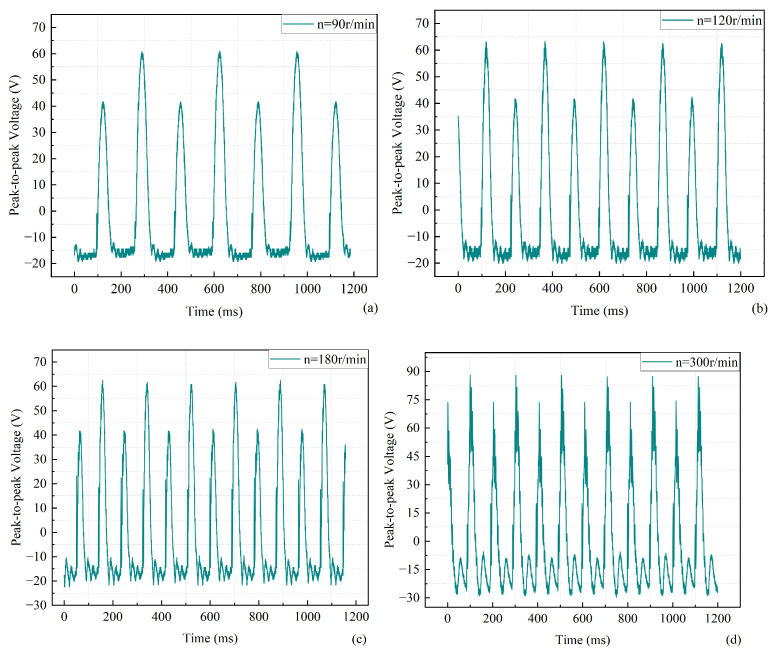
Output voltage versus time for a baffle size of 10 mm and 10 mm. (**a**) 90 r/min. (**b**) 120 r/min. (**c**) 180 r/min. (**d**) 300 r/min.

Three sets of baffles with different sizes were selected to verify whether different baffle sizes affect the output voltage of the piezoelectric energy harvester. The performance of the mechanism was analyzed experimentally. The dimensions of the three sets of baffles were 10 mm and 10 mm, 20 mm and 20 mm, and 10 mm and 35 mm. The relationship between the output voltage and time when the baffle sizes are 10 mm and 10 mm is shown in Figure 7. The waveform plots corresponding to some of the velocities in the experiment can show the trend of the overall experimental results. During the experiment, the output voltage of the energy harvester first decreases and then increases as the speed increases. However, the experimental results show an increase in the overall output voltage of the energy harvester. The output peak-to-peak voltage of the energy harvester was about 88.8 V at a speed of 180 r/min and about 115 V at a speed of 300 r/min. Also, the phenomenon of different heights of adjacent peaks in each waveform graph needs to be noted. This is because the deformation caused by the self-vibration of the piezoelectric beam is not as great as the deformation generated by the rotor collision. The piezoelectric beam has a self-vibrating space when the rotor contact is disengaged each time. Therefore, high and low peaks are always present in phases.

**Figure 8:**
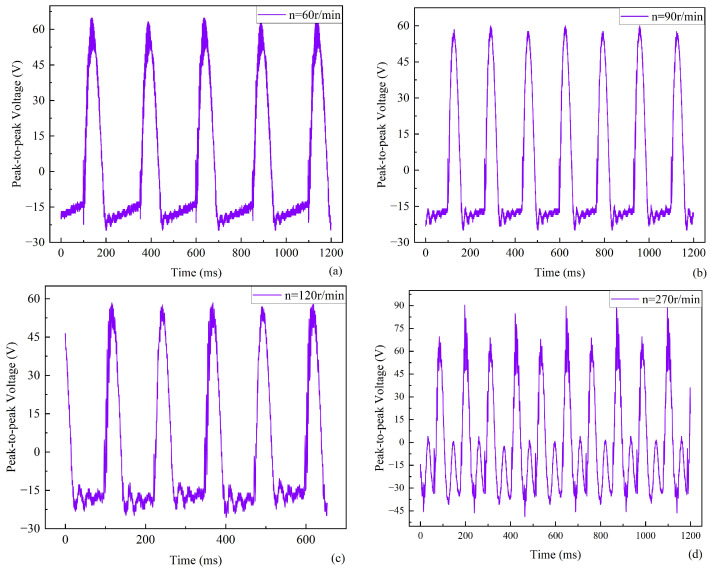
Output voltage versus time for a baffle size of 20 mm and 20 mm. (**a**) 60 r/min. (**b**) 90 r/min. (**c**) 120 r/min. (**d**) 270 r/min.

The relation between the output voltage and time is shown in Figure 8 when the baffle sizes are 20 mm and 20 mm. As the speed increases, the output voltage of the harvester also increases, as can be seen from the selected curve of speed versus time. The output peak-to-peak voltage of the energy harvester is about 100 V when the speed is 180 r/min, and the output peak-to-peak voltage of the energy harvester is about 142 V when the speed is 240 r/min. Furthermore, there is a clear result that the average output voltage of the piezoelectric energy harvester under these conditions is higher than that of the energy harvester with baffle sizes of 10 mm and 10 mm. This illustrates that the performance of the piezoelectric energy harvester is favorably improved by increasing the baffle size. Figure 8 shows that the number of voltage peaks increases with speed, accompanied by the appearance of several small wavelets. One possible reason for this is that the rotor impact frequency is close to the intrinsic frequency of the piezoelectric beam. Multiple self-vibrations of the piezoelectric beam are generated after a single impact of the rotor, and the self-vibration deformation gradually decreases. When the difference between the excitation frequency and the resonance frequency increases, resonance cannot be triggered, the deformation amplitude decreases, and the signal waveform becomes smoother, as shown in Figure 8a.

The frequency *f_b_* = 23.8 Hz is obtained by substituting the relevant data and characteristics in Table 1 into Equation (14). The rotor with two contacts can generate two excitation signals per full rotation. Three response points can occur for one excitation signal due to the rigid connection of three piezoelectric beams. The frequency of the rotor with two contacts is 3.97 r/s, i.e., 238.2 r/min. Therefore, the superior output performance of the piezoelectric energy harvester is obtained when the speed is 238.2 r/min for the rotor with two contacts. Experiments show that good output can be obtained at 240 r/min. The theoretical results are close to the experimental results. Output performance is optimized when the driving frequency approximates the resonant frequency.

**Figure 9:**
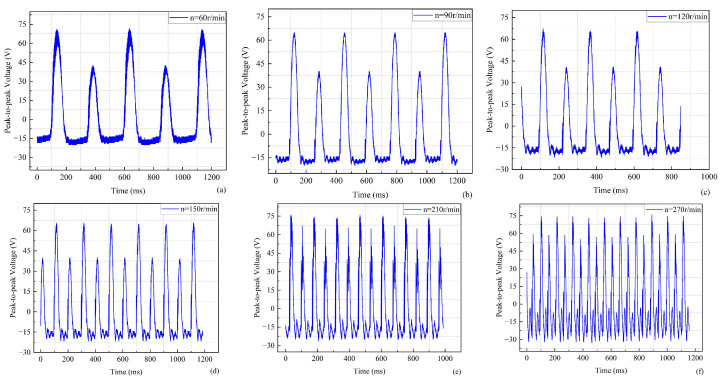
Output voltage versus time for a baffle size of 10 mm and 35 mm. (**a**) 60 r/min. (**b**) 90 r/min. (**c**) 120 r/min. (**d**) 150 r/min. (**e**) 210 r/min. (**f**) 270 r/min.

The relation between the output voltage and time when the baffle sizes are 10 mm and 35 mm is shown in Figure 9. From the experimental results, the peak-to-peak output voltage of this piezoelectric energy harvester is higher at a speed of 60 r/min. The output voltage of the energy harvester first decreases and then increases with the continuous increase of the rotational speed. The output voltage shows a hump-like characteristic in the range of 60–300 r/min. Comparing baffles of sizes 20 mm and 20 mm, asymmetric baffles will result in uneven stiffness distribution, thereby causing slight deviations in resonance frequencies. This will broaden the frequency band, but reduce the peak output during resonance. Therefore, a conclusion can be drawn from the experimental results: The bandwidth of the piezoelectric energy harvester can be increased when the dimensions of the two baffles differ significantly.

The baffle acts as a force transfer medium between the three cantilever beams. Different sizes will affect the stiffness matching between the beams and the uniformity of the force distribution. A larger and symmetrical baffle can more effectively transfer the rotor’s impact force to the three beams, avoiding local force concentration or force loss. Three sets of experimental results show that the proposed rigid parallel-connected piezoelectric energy harvester can meet the demand for capturing energy at low or high speeds, and it is more efficient in capturing energy at high speeds. Additionally, the structures with multiple beams can interact with each other, and there is a possibility of internal resonance. Internal resonance can broaden the frequency band for acquisition. Different matching sizes of baffles also have a subtle effect on the performance of the energy harvester. The peak-to-peak voltage output of the energy harvester is more stable at a rotational speed of 240 r/min. Therefore, the optimal speed of the rotor should be determined as 240 r/min.

**Figure 10:**
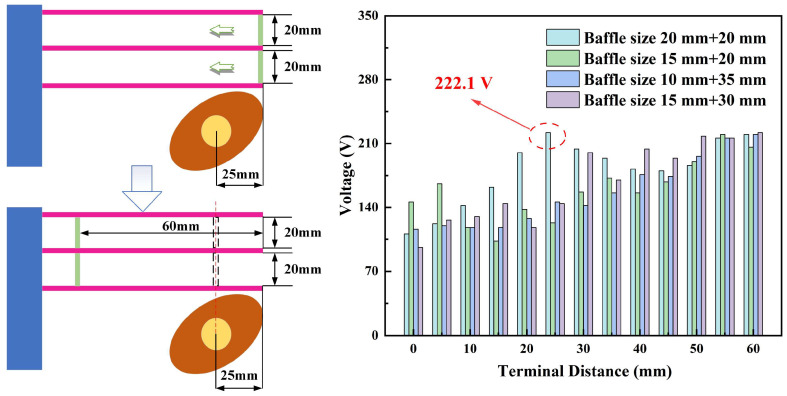
Output voltage versus time with baffles symmetrically arranged at different positions.

Four sets of baffle sizes were chosen for the experiment to verify whether the different mounting positions of the baffles affect the efficiency of the piezoelectric energy harvester. The selected baffle sizes were 20 mm and 20 mm, 15 mm and 20 mm, 10 mm and 35 mm, and 15 mm and 30 mm. The output voltage versus time for an energy harvester with symmetrically arranged baffles at different positions is shown in Figure 10. Baffle A and baffle B were moved simultaneously to the left for the test, by 5 mm each time. The constant speed of the selected rotor was 240 r/min. The energy collector shows fluctuations in voltage when the distance between the baffle and the cross-section of the free end of the piezoelectric beam is 20 mm to 35 mm. The vertical line where the rotor axis is located is approached by the baffle, which is one possible reason for the fluctuations. However, the point where the energy harvester reaches high voltage varies with the different sizes of the baffle. The results from the four sets show that the overall output voltage of the piezoelectric energy harvester increases as the baffles move to the left. The most suitable position for the baffle should be arranged near the vertical line where the rotor axis is located. The position of the baffle determines the force arm acting on the cantilever beam. When the baffle is close to the rotor axis, it achieves the maximum effective force on the beam.

**Figure 11:**
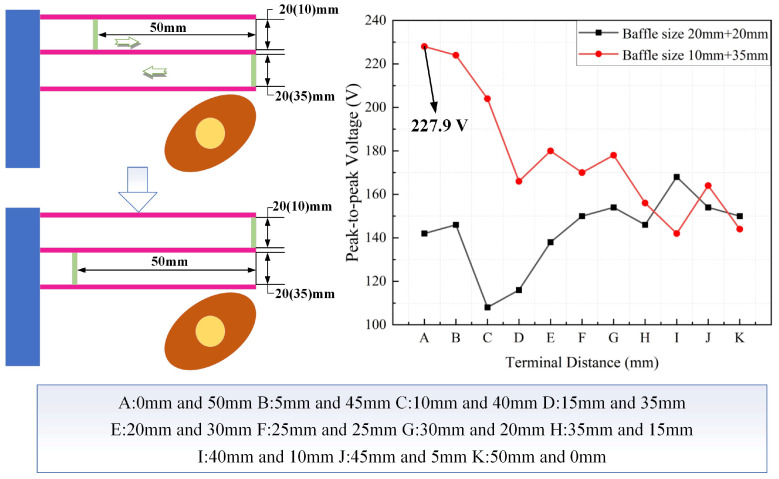
Output voltage versus time with baffles asymmetrically arranged at different positions.

The output voltage versus time with baffles asymmetrically arranged at different positions is shown in Figure 11. To verify whether the asymmetric arrangement of the baffles has an effect on performance, the distance between the initial position of baffle A and the free end of the piezoelectric beam was set at 50 mm. Baffle A was moved to the right by 5 mm each time, and the end position was aligned with the starting position of baffle B. The distance between the initial position of baffle B and the free end of the piezoelectric beam was 0 mm. Baffle B was moved to the left 5 mm at a time and the end position was aligned with the starting position of baffle A. The constant speed selected throughout the test was 240 r/min. The experimental results show that the output voltage of the energy harvester with baffle sizes of 10 mm and 35 mm shows a decreasing trend. By contrast, the output voltage of the energy harvester with baffle sizes of 20 mm and 20 mm shows a trend of first decreasing and then increasing. The performance of the energy harvester remains almost unchanged when baffle A is near the right end and baffle B is near the left end. A possible reason for this is that the impact force of the rotor on piezoelectric beam C is transferred to piezoelectric beam A and piezoelectric beam B through the baffles. The force arm where piezoelectric beam C is located increases with the movement of the baffle B. The force on piezoelectric beam B decreases due to the longer force arm.

The experimental results in Figure 10 and Figure 11 illustrate that different arrangements and positions of the baffles affect the output voltage. The different sizes of the baffles cause changes in the force applied to the three piezoelectric beams A, B and C. Therefore, the output performance of the piezoelectric energy harvester shows different characteristics. The energy collector performs better when the sizes of the two baffles are both 20 mm. The performance of the energy collector is better when the arrangement of the baffle is located at or near the vertical line where the rotor is located.

**Figure 12:**
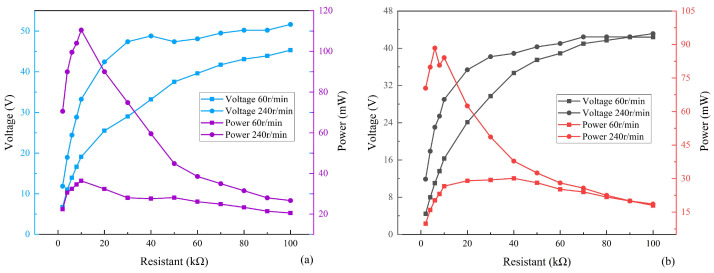
Output power under different conditions. (**a**) Baffle size: 20 mm and 20 mm. (**b**) Baffle size: 10 mm and 35 mm.

Experiments with two pairs of energy harvesters under different loads are shown in Figure 12 to more intuitively demonstrate the performance of the piezoelectric energy harvester. To calculate the power, Equation (17) was adopted due to the difficulty in obtaining the internal resistance of the piezoelectric material. *R*_0_ represents the external load and *V* represents the voltage obtained from the measurement. The voltage and power curves of the piezoelectric energy harvester with baffle sizes of 20 mm and 20 mm are shown in Figure 12a. At this time, the RMS voltage and the average power of the piezoelectric energy harvester at a load of 10 kΩ and a speed of 240 r/min are 33.24 V and 110.49 mW, respectively. The average power of the energy harvester at a load of 10 kΩ and a speed of 60 r/min is 36.44 mW. As the external load impedance increases, the voltage across the external load gradually increases and eventually converges to the open-circuit voltage value of 50.2 V for this device. Meanwhile, the maximum output power of the system gradually increases as the external load impedance increases. When the external load impedance reaches a certain value, the maximum output power gradually decreases as the external load impedance increases. The optimal load impedance corresponding to the peak power is 10 kΩ.

The power curves of the piezoelectric energy harvester with baffle sizes of 10 mm and 35 mm are shown in Figure 12b. The average power of the piezoelectric energy harvester at a load of 10 kΩ and a speed of 240 r/min is 84.10 mW. The average power of the energy harvester at a load of 40 kΩ and a speed of 60 r/min is 30 mW. The results further show that the proposed rigid parallel piezoelectric energy harvester can achieve high power output under both high- and low-speed conditions. The size of the baffle also has a different effect on the power of the energy harvester. The 20 mm and 20 mm baffle sizes are obviously more suitable for the piezoelectric energy harvester to collect energy at low speeds and also achieve higher power output at high speeds. Therefore, the dimensions of the rigid connectors obtained in the other experiments in this work—20 mm and 20 mm—are reasonable.

A quantitative comparison was conducted between the theoretical and experimental results of the output voltage and power of the piezoelectric energy harvester. The 20 mm and 20 mm baffle sizes were selected, with a load of 10 kΩ. The theoretical values of the output voltage and power under two different rotational speeds of 60 r/min and 240 r/min were calculated and compared with the experimental results, as presented in Table 3.

The main sources of error are that in the theoretical model, the mechanical damping was assumed to be a constant value, while in the experiment, the damping slightly fluctuated due to the influence of assembly clearance. The theoretical calculation of the impact force adopts an average value, while in the experiment, there is fluctuation in the contact force between the rotor and the beam. However, the comparison results have initially verified the consistency between the theoretical and experimental results.

**Figure 13:**
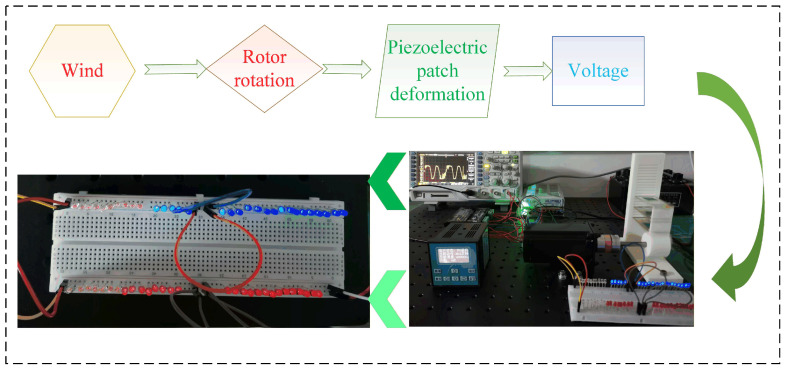
Application experiment of the piezoelectric energy harvester.

The experiment of lighting the LED is shown in Figure 13 to illustrate the good practical applicability of the piezoelectric energy harvester. The experimental results show that the proposed piezoelectric energy harvester can light up about sixty LEDs. Therefore, the energy harvester has some application value and can be considered for use in some outdoor micro-power consumption devices in the future. For example, the energy harvester can be used to harvest wind energy. Small vertical-axis wind turbines used on urban rooftops, in rural areas, or on offshore platforms operate at 20–80 r/min under low wind speeds. The rotational motion of the turbine blades provides continuous excitation for the piezoelectric energy harvester. The harvested energy can power wind speed sensors, direction controllers, or wireless data transmission modules. In general, the energy generated by the piezoelectric energy harvester can meet the energy supply needs of some micro and small devices. Therefore, the proposed energy harvester possesses promising application prospects. The results of the comparison of this work with the work of other scholars are shown in Table 4. Although the application conditions are inconsistent, the output performance of the prototype of the present work has some advantages in the same area or volume of piezoelectric ceramics.

## 5. Conclusions

This work presents a cantilevered piezoelectric energy harvester based on an adjustable rigid parallel connection for capturing rotational motion. The theoretical model of the structure was established, equations for voltage and power were derived, and experiments were conducted by varying the baffle size, the baffle position relative to the free end, and the number of rotor bumps. The key conclusions are outlined as follows:

(1) Different arrangements and positions of the baffles affect the output voltage. The energy harvester performs better when the sizes of the two baffles are both 20 mm. Additionally, the efficiency of the energy harvester can also be further improved when the baffle arrangement is close to the vertical line of the rotor axis.

(2) The proposed piezoelectric energy harvester can output high power at both high and low rotational speeds. The maximum average power of the energy harvester is 110.49 mW at a rotational speed of 240 r/min and an external load of 10 kΩ. The maximum average power of the energy harvester was 36.44 mW at a rotational speed of 60 r/min and an external load of 10 kΩ.

(3) The rigid parallel connection of multiple piezoelectric beams effectively enhances the overall performance of the harvester.

A limitation of this study is that it only tests specific variables (baffle size, baffle position, number of rotor bumps) without exploring the influence of other factors (e.g., material properties of the baffle or application scenarios). Future research will expand the range of variables, optimize the structural design to achieve broader speed adaptability, and verify the harvester’s long-term stability in practical rotational motion scenarios.

## Figures and Tables

**Table 1:** Material parameters.

Materials	Parameters	Value
Piezoelectric ceramic (PZT)	Young’s modulus (N/m^2^)	5.6 × 10^10^
	Poisson ratio	0.36
	Density (kg/m^3^)	7500
	Piezoelectric coefficient e_33_ (C/N)	670 × 10^−12^
	Piezoelectric coefficient e_31_ (C/N)	−186 × 10^−12^
	Dielectric constant ε_33_/ε_11_	3400/3130
	Length (mm)	60
	Width (mm)	31
	Thickness/height (mm)	0.2
Baffle	Length (mm)	5
	Width (mm)	30
	Thickness/height (mm)	10/15/20/25/30/35
Copper	Young’s modulus (N/m^2^)	11.2 × 10^10^
	Poisson ratio	0.35
	Density (kg/m^3^)	8780
	Length (mm)	80
	Width (mm)	33
	Thickness/height (mm)	0.3
	Young’s modulus (N/m^2^)	11.2 × 10^10^

**Table 2:** Supplementary parameters.

Equations	Parameters	Value
Equation (13)	Equivalent resistance of the piezoelectric ceramic (kΩ)	10
	Piezoelectric ceramic capacitance (nF)	280
	Electromechanical coupling coefficient of the piezoelectric ceramic (μN/V)	16.6
Equation (15)	Exciting force generated by the rotating the motor (N)	5–25
Equation (17)	Load resistance (kΩ)	0–100
	Output voltage (V)	0–50.2

**Table 3:** Comparison of theoretical value and experimental results.

Motor Speed	Theoretical Value	Experimental Result	Relative Error
60 r/min	19.75 V	19.1 V	3.29%
	39.01 mW	36.44 mW	6.59%
240 r/min	33.6 V	33.24 V	1.07%
	112.9 mW	110.49 mW	2.13%

**Table 4:** Comparisons between this work and some existing works.

	Piezoelectric Patch Number	Piezoelectric Patch Size	RMS Voltage	Power
Yang et al. [17]	1	45 × 8 × 0.4 mm^3^	14.14 V	50 μW
Usha et al. [18]	1	76.5 × 25 × 0.5 mm^3^	19.67~3.996 V	12.35–1.313 mW
Li et al. [20]	1	20 × 10 × 0.2 mm^3^	6.3 V	0.25 mW
He et al. [25]	1	60 × 20 × 0.3 mm^3^	24.84 V	15.425 mW
This work	1	60 × 31 × 0.2 mm^3^	33.24 V	36.83 mW

## Data Availability

The original contributions presented in the study are included in the article. Further inquiries can be directed to the corresponding author.

## References

[1] Zhou X., Wu S., Wang X., Wang Z., Zhu Q., Sun J., Huang P., Wang X., Huang W., Lu Q. (2024). Review on piezoelectric actuators: Materials, classifications, applications, and recent trends. Front. Mech. Eng..

[2] Sawane M., Prasad M. (2023). MEMS piezoelectric sensor for self-powered devices: A review. Mater. Sci. Semicon. Process..

[3] Abdulkadir C.A., Oğuzhan Ç. (2023). Piezoelectric materials in civil engineering applications: A review. ACS Omega.

[4] Ali A., Ali S., Shaukat H., Khalid E., Behram L., Rani H., Altabey W.A., Kouritem S.A., Noori M. (2024). Advancements in piezoelectric wind energy harvesting: A review. Results Eng..

[5] Liu L., He L., Han Y., Zheng X., Sun B., Cheng G. (2023). A review of rotary piezoelectric energy harvesters. Sens. Actuators A Phys..

[6] Megdich A., Habibi M., Laperrière L. (2025). Review of progress in 4D printing of piezoelectric energy harvesters. Mater. Sci. Eng. R..

[7] Xian W., Lee S. (2024). A pendulum based frequency-up conversion mechanism for vibrational energy harvesting in low-speed rotary structures. J. Intell. Mater. Syst. Struct..

[8] Zhang L., Kan J., Lin S., Liao W., Yang J., Liu P., Wang S., Zhang Z. (2024). Design and performance evaluation of a pendulous piezoelectric rotational energy harvester through magnetic plucking of a fan-shaped hanging composite plate. Renew. Energy.

[9] Han Y., Wang C., Sun L., Wang H., Yang B., He L. (2024). Research on a rotary piezoelectric energy harvester based on movable magnets. Smart Mater. Struct..

[10] Xia C., Tang L., Meng T., Wang J., Yin P., Zhang D., Li Z., Aw K.V. (2025). A V-shaped galloping piezoelectric energy harvester exploiting bending and torsional modes. Int. J. Mech. Sci..

[11] Liu M., Xia H., Xia D., Liu G.Q. (2021). Research on frequency bandwidth and phase difference of piezoelectric resonant cantilever based on mass. Microsyst. Technol..

[12] Ma X., Gong L., Zeng Z., Wu Y., Guo W., Chen H., Feng G. (2025). Design and optimization of a compact broadband piezoelectric energy harvester system with enhanced efficiency. Renew. Energy.

[13] He L.P., Wang Z., Yu G., Shen Z.Y., Jiang S., Cheng G.M. (2022). Design and experimental research of magnetically excited rotating piezoelectric energy harvester. Microsyst. Technol..

[14] Hong Y., Sui L., Zhang M.Y., Shi G.C. (2018). Theoretical analysis and experimental study of the effect of the neutral plane of a composite piezoelectric cantilever. Energy Convers. Manag..

[15] Xie X.D., Carpi A., Wang Q. (2017). A theoretical model for a piezoelectric energy harvester with a tapered shape. Eng. Struct..

[16] Yao Z., Li C. (2025). A rotary piezoelectric electromagnetic hybrid energy harvester. Micromachines.

[17] Yang B., Pavel T.F., Zdenek H.D., Jan S.L., Petr L.S., Pavel S.D., Robert M.K. (2018). Investigation of a cantilever structured piezoelectric energy harvester used for wearable devices with random vibration input. Mech. Syst. Signal Process..

[18] Usharani R., Uma G., Umapathy M., Choi S.B. (2017). A new piezoelectric-patched cantilever beam with a step section for high performance of energy harvesting. Sens. Actuators A Phys..

[19] Raju S.S., Choi S.B., Umapathy M., Uma G. (2019). An effective energy harvesting in low frequency using a piezo-patch cantilever beam with tapered rectangular cavities. Sens. Actuators A Phys..

[20] Li X., Ming Z., Xiao M., Yang K., Ma Y.K., Luo Y. (2019). A wide-band piezoelectric harvester based on cantilever beam. Ferroelectrics.

[21] Chen L.H., Xue J.T., Pan S.Q., Chang L.Q. (2020). Study on cantilever piezoelectric energy harvester with tunable function. Smart Mater. Struct..

[22] Xie Z.Q., Zhou S.X., Xiong J.T., Huang W.B. (2019). The benefits of a magnetically coupled asymmetric monostable dual-cantilever energy harvester under random excitation. J. Intell. Mater. Syst. Struct..

[23] Tang L.P., Wang J.G. (2018). Modeling and analysis of cantilever piezoelectric energy harvester with a new-type dynamic magnifier. Acta. Mech..

[24] Liu L., He L.P., Liu X.J., Han Y.H., Sun B.Y., Cheng G.M. (2022). Design and experiment of a low frequency non-contact rotary piezoelectric energy harvester excited by magnetic coupling. Energy.

[25] He L.P., Wang Z., Wu X.Q., Zhang Z., Zhao D., Tian X.C. (2020). Analysis and experiment of magnetic excitation cantilever-type piezoelectric energy harvesters for rotational motion. Smart Mater. Struct..

[26] Kan J., Zhang L., Zhang Y.Z. (2023). Development and performance evaluation of a wheel-type cantilevered piezoelectric rotational energy harvester via an unfixed exciting magnet. J. Intell. Mater. Syst. Struct..

[27] Liu X.J., Yan Y.F., Zhong F., Yang J.W., Zhang L.M., He L.P. (2024). Pendulum type magnetically coupled rotary piezoelectric energy harvester. Smart Mater. Struct..

[28] Heidari M., Rahimi G.H., Bab S. (2024). Ocean non-linear energy harvesting (NEH) with a buckled piezoelectric beam. Appl. Ocean Res..

[29] Su D., Sun C., Wang L. (2024). Design and evaluation of a piezoelectric-electromagnetic energy harvester with a lever structure. Mech. Syst. Signal Process..

[30] Gao Y.J., Leng Y.G., Fan S.B., Lai Z.H. (2014). Performance of bistable piezoelectric cantilever vibration energy harvesters with an elastic support external magnet. Smart Mater. Struct..

[31] Guan Q.C., Ju B., Xu J.W., Liu Y.B., Feng Z.H. (2013). Improved strain distribution of cantilever piezoelectric energy harvesting devices using H-shaped proof masses. J. Intell. Mater. Syst. Struct..

[32] Wang Z., He L.P., Zhang Z., Zhou Z.M., Zhou J.W., Cheng G.M. (2021). Research on a piezoelectric energy harvester with rotating magnetic excitation. J. Electron. Mater..

